# Ladies’ Hospital Association

**Published:** 1870-06

**Authors:** 


### Ladies' Hospital Association.

It will be welcome news to the profession of this city and surrounding country the announcement that the organization named in the caption of this article, is now in successful operation. The need of an hospital devoted exclusively to the medical and surgical treatment of the diseases of females has long been felt, and now, thanks to the ladies of this city, an institution of this nature is *au fait accompli.*

The Board of Directory is composed of ladies who, in entering upon this grand work, have evinced an energy worthy of them.

It could not be expected, during the initiatory steps of a work of this kind, where much money would be required in order to carry out the purposes ultimately aimed at, that an edifice could at once be erected with sufficient capacity to accommodate a large number of patients, therefore the ladies have wisely entered into an arrangement with the trustees of the Buffalo General Hospital—the latter assigning to the former a section of the hospital, consisting of general and private wards, the patients to be under the medical and surgical treatment of the staff of the General Hospital, the Ladies' Hospital Association paying into the treasury of the General Hospital five dollars per week for each patient received. Under this financial arrangement whoever contributes funds toward the support of one of the institutions, contributes also, indirectly, to the support of the other; the resulting pecuniary benefits to both being mutual and reciprocal, while in their relation to the care of the sick and unfortunate they are entirely distinct, the one from the other, except that both organizations are under the supervision of the able and faithful Superintendent, Mr. Wm. C. Bagley.

I should not fail to state that the Ladies' Hospital Association embraces a Lying-in Department, and several lying-in women can already testify to the beneficent provisions here made for their care and comfort.

I have thought some record should be made of this new institution, which is in fact, if not in name, a "Woman's Hospital," and know of no more suitable place for record than the pages of the *Journal*, I append the list of officers and members of the Executive Committee. These names give ample assurance and guarantee of the success of the enterprise:

*President.*—Mrs. J. B. Skinner.

*First Vice President.*—Mrs. Thomas F. Rochester.

*Second Vice President.*—Mrs. J. F. Chapman.

*Third Vice President.*—Mrs. E. Young.

*Executive Committee.*—Mrs. R. J. Sherman, Mrs. C. F. Wadsworth, Mrs. W. C. Robinson, Mrs. J. N. Scatcherd, Mrs. S. D. Sykes.

Executive Committee meet every Tuesday, at 10 A. M., and the association meet the second Tuesday in each month, at three P. M., at the Hospital.

